# Reducing non-sterile glove use in a sexual health and HIV department: A quality improvement project to address clinical practices

**DOI:** 10.1177/09564624251326696

**Published:** 2025-03-13

**Authors:** Laurie Smith, Amanda Clarke, Gillian Dean

**Affiliations:** 1 12190 Brighton & Sussex Medical School (BSMS), Brighton, UK; 2 Brighton and Hove Sexual Health and Contraception Service, University Hospitals Sussex NHS Trust, Brighton, UK

**Keywords:** Sustainability, environment, non-sterile gloves, education

## Abstract

**Background:**

Climate change is a huge public health threat, necessitating reductions in carbon emissions, particularly from single-use plastics like non-sterile gloves (NSG). This quality improvement project aims to explore whether use of targeted educational material changes staff attitudes towards NSG use in clinical practice within a Sexual Health and HIV department.

**Methods:**

A pre-intervention survey was circulated to all clinicians. Subsequently, various methods encouraged appropriate NSG use including video guidance of performing venepuncture without gloves in line with Trust policy, educational presentations, and patient-facing posters for waiting rooms. NSG procurement data were obtained, and a post-intervention survey evaluated whether NSG use had changed following the interventions.

**Results:**

Sixty-three percent of staff believed they had reduced their personal glove use in the past year. Many staff believed the best way to reduce inappropriate glove use was through education as well as empowering patients through posters. Glove procurement data comparing 2023/2024 to pre-COVID 2019/2020 showed a 45.2% decrease in NSG orders from 173,110 to 94,800 per year.

**Conclusions:**

Staff education is successful in reducing inappropriate NSG use, with patient posters and targeted staff presentations the most effective measures to drive behaviour change and therefore reduce NSG use.

## Introduction

Climate change is the biggest threat to public and global health in the 21^st^ century,^1^ and the National Health Service (NHS), responsible for nearly 5% of the UK’s carbon footprint,^2^ has a significant role in reducing its environmental impact. One challenge to achieving net-zero emissions is single-use plastic, particularly non-sterile gloves (NSG). The NHS in England uses 3.8 million NSG every day,^3^ contributing to carbon emissions and financial costs. NSG may be inappropriately used (used when there is no clinical indication i.e. no risk of bodily fluid transmission) in nearly 60% of procedures.^4^ Inappropriate use of gloves may increase the risk of the spread of transmissible infections between patients when gloves are used in place of handwashing.

A single NSG has a carbon footprint of 26g of carbon dioxide equivalent (CO_2_e),^5^ leading to a total daily carbon footprint of NSG in the NHS of 98.8 million grams of CO_2_e. There are also ethical issues: gloves are often produced by migrant workers under poor working conditions in Malaysia using large quantities of oil.^6^ Reducing inappropriate glove use requires behaviour change from staff.

## Aims

This project investigates whether use of targeted educational material changes staff attitudes towards NSG use in clinical practice in a Sexual Health and HIV department. This was measured by a pre- and post-intervention survey and procurement data analysis. The secondary aim was to improve staff awareness of appropriate NSG use, thereby reducing the number of gloves procured.

## Methods

### Pre-intervention survey – Spring 2023

Prior to this project, an online survey was circulated to clinical staff (doctors, nurses, health advisors and clinical support workers (CSW)) in the department to assess glove use and related concerns, with 76 staff members participating (86% return rate). The survey combined quantitative and qualitative data using a Likert scale and free text. The results were shared with staff in July 2023.

### Educational tool and video – November 2023

An educational tool was presented to the department which reviewed appropriate glove use in line with Trust policy, and addressed staff concerns from the previous survey. A video demonstrating how correct use of the butterfly self-sheathing needle when taking blood without gloves in line with updated Trust policy, can eliminate blood-borne virus (BBV) transmission risk from needlestick injuries.

### Patient information – January 2024

An online poster displayed on television screens in patient waiting rooms showed the carbon emissions saved from reduced NSG use in the department from 2019 to 2023.

### Post-intervention survey – February 2024

A follow-up voluntary survey was circulated to the clinical staff members to evaluate changes in glove use following interventions. The survey mainly featured questions from the first survey.

### Glove usage data collection – March 2024

Glove procurement data comparing 2023/2024 to pre-COVID 2019/2020 were obtained to measure changes in glove orders post-intervention.

### Patient information – April 2024

A digital information screen was produced with the Clinical Media Centre to outline to patients that staff may not be wearing gloves when, for example, taking blood, giving intramuscular injections, handing out medication or examining the skin.

Ethical approval was not required for this quality improvement project due to the anonymous optional staff survey. Consent was assumed if the survey was completed. This project was reported in line with the Standards for Quality Improvement Reporting Excellence guidelines.^7^

## Results

**Figure 1.**
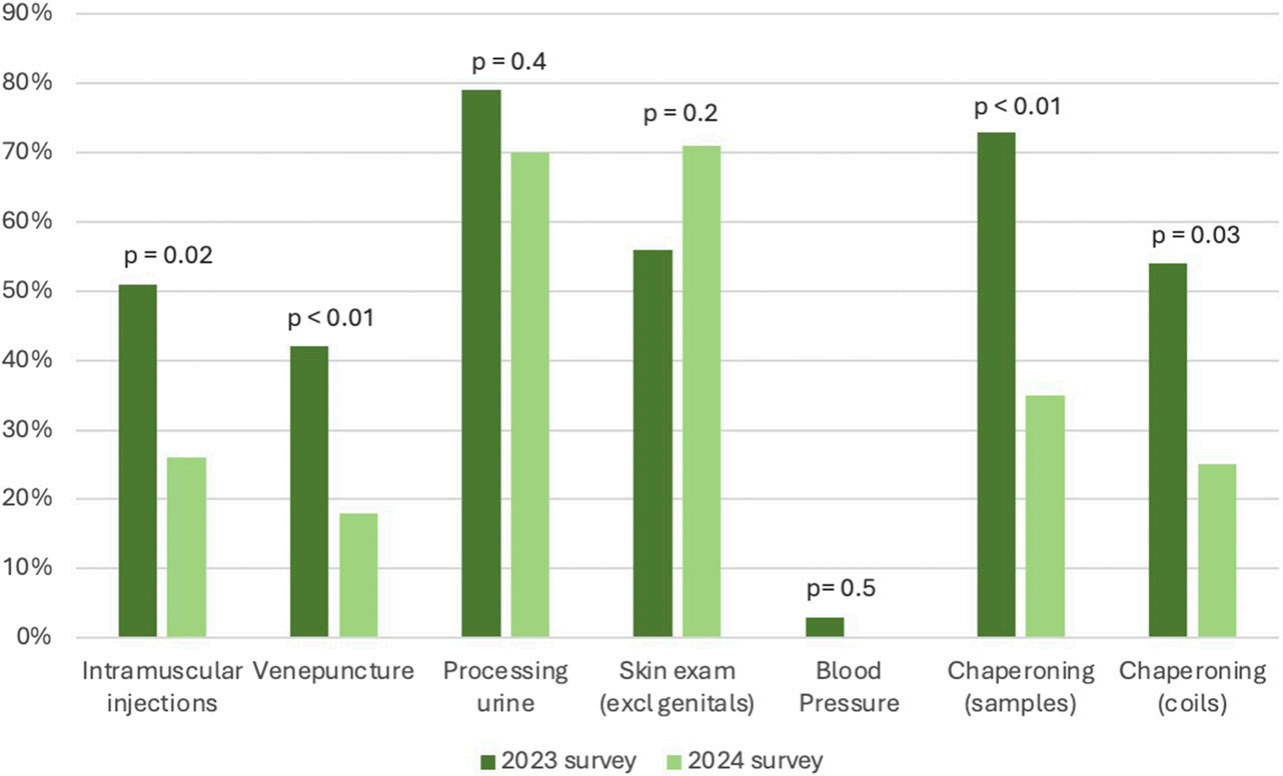
Percentage of staff self-reporting NSG use for different clinical tasks. Data from the 2024 post-intervention survey is compared to the 2023 pre-intervention survey. N.B. 0% of staff wore gloves for blood pressure in the 2024 survey.

Of the 89 clinical members of staff, 50 completed the post-intervention survey, (56% response rate). This represented 24 nurses (48%), 17 doctors (34%), 5 health advisors (10%), and 4 CSW (8%). Responses on glove use for routine clinical tasks were compared to the 2023 survey (see Figure 1). All clinical tasks, except skin examination (which saw a 26.8% increase) saw a reduction in inappropriate glove use. The decreases ranged from an 11.4% (processing urine) to 57.1% (venepuncture), with statistically significant reductions for intramuscular (IM) injections, venepuncture, and chaperoning both coils and samples.

A Likert scale showed increased staff confidence in performing IM injections and venepuncture without gloves: 72% felt extremely confident in venepuncture, compared to 60.5% in 2023, and 59.5% felt extremely confident in IM injections, compared to 47% in 2023. Regarding glove use habits over the past year, 62.5% of staff reported a decrease in glove use following interventions, while 35.4% said it remained the same, and 2.1% noted an increase.

Staff identified educational presentations and patient-facing posters as the most effective interventions to reduce NSG use, while sustainability e-learning was deemed least effective. Free text boxes showed enthusiasm for reducing consumption of single-use plastics further, however barriers included a preference to wear gloves for ‘intimate’ procedures like gluteal injections, fear of exposure to patient blood, drawing up antibiotics, and lack of clear information on when and when not to wear gloves. Other barriers contributing to unnecessary glove use included perceived peer pressure with newer, younger recruits presuming more experienced colleagues expect them to use gloves and could risk being criticised if they do not.

Departmental glove procurement data showed a 45.2% decrease in annual NSG orders, from 173,110 in 2019–2020 to 94,800 in 2023–2024. This represented a financial saving of over £1200. Procurement data were not measured in the years 2020–2023 due to non-demand-based PPE stock being supplied by the Department of Health during the coronavirus pandemic. This made the exact quantity of additional NSG received in these years difficult to measure.

## Discussion

Targeted education successfully improved healthcare workers’ (HCWs) glove use practice, particularly for venepuncture, which was already low at baseline (42%). This was explained by the department’s long-term commitment to sustainable practice and having ‘green leaders’ to encourage behaviour change through role-modelling and education.

The highest rate of glove use was for skin-to-skin contact during examination (excluding genitals), potentially due to increased scabies and infectious syphilis cases seen in 2024. This behaviour may also indicate a tendency among staff to attempt to depersonalise patient interactions, as HCWs may use gloves as a physical barrier against perceived patient uncleanliness,^4^ despite handwashing being equally effective. Addressing these deep-seated emotions may help reduce glove use, though some staff may resist changes that challenge their existing belief system.

Additionally, some staff feel unable to challenge others, or defend themselves, as wearing gloves is a personal choice,^8^ with some staff fearing being accused what they are doing is wrong, both by patients and colleagues.^8^ Habitual glove use, reinforced by the mandatory COVID-19 PPE protocols,^5^ may also play a role.

The 45% reduction in glove use saved 2036 kg of CO2e, equivalent to driving over 7200 miles in a petrol car.^9^ Staff confidence likely improved due to understanding that gloves do not impact needlestick injury management and HIV cannot be transmitted in the 99% of patients who are on effective antiretroviral treatment.^10^ With correct use of blood-taking equipment, such as the butterfly self-sheathing needle, the risk of such injuries is effectively zero. The increase in confidence gained in this department, which is notable considering the higher proportion of patients with BBVs, suggests this expertise could be valuable for other areas.

## Strengths

This project examined HCW attitudes regarding glove use before and after specific interventions. Through repeating parts of the pre-intervention survey, we were able to directly compare staff thoughts and confidence pre- and post- education. Use of a mixed-methods survey, combining both qualitative and quantitative data provided a deeper understanding of the reasons for staff behaviour change. Through gathering HCW concerns via free text boxes in the pre-intervention survey, barriers to reducing glove use were directly addressed. Staff feedback on which proposed interventions they thought would be successful could be useful in guiding future efforts to expand the project across the wider NHS.

## Limitations

Post-intervention survey completion (56%) was lower than pre-intervention (84%), possibly due to questionnaire fatigue. The survey results may not represent views across other healthcare systems, given this department had good baseline knowledge and had already implemented sustainability initiatives. Extrapolating to other departments may be less successful, particularly if staff are less concerned about the environmental and ethical impacts of glove use.

Comparing direct pre- and post- intervention glove procurement data would have been useful, but changes in procurement methods during the coronavirus pandemic precluded this. Furthermore, staff turnover between the two surveys may have influenced results, making it unclear whether shifts in attitudes reflect genuine changes among existing staff or the inclusion of perspectives from new team members. Additionally, a longer post-education period for change to embed may have yielded stronger results.

## Conclusion

Staff education effectively reduced inappropriate glove use particularly for venepuncture and IM injections. If expanded, there could be significant financial savings, less single-use plastic, and reduced carbon emissions, helping the NHS reach its net-zero target.

To optimise resources and reduce waste across the NHS, future efforts should involve staff opinions from other clinical areas, particularly those without prior knowledge of carbon-saving initiatives. Patient posters and staff presentations were identified as the most effective interventions, though multiple interventions may be needed to drive lasting behaviour change and reduce inappropriate non-sterile glove use.
